# Robustness and Information Propagation in Attractors of Random Boolean Networks

**DOI:** 10.1371/journal.pone.0042018

**Published:** 2012-07-30

**Authors:** Jason Lloyd-Price, Abhishekh Gupta, Andre S. Ribeiro

**Affiliations:** Laboratory of Biosystem Dynamics, Computational Systems Biology Research Group, Department of Signal Processing, Tampere University of Technology, Tampere, Finland; Technical University of Madrid, Italy

## Abstract

Attractors represent the long-term behaviors of Random Boolean Networks. We study how the amount of information propagated between the nodes when on an attractor, as quantified by the average pairwise mutual information (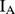), relates to the robustness of the attractor to perturbations (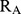). We find that the dynamical regime of the network affects the relationship between 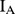 and 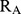. In the ordered and chaotic regimes, 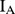 is anti-correlated with 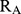, implying that attractors that are highly robust to perturbations have necessarily limited information propagation. Between order and chaos (for so-called “critical” networks) these quantities are uncorrelated. Finite size effects cause this behavior to be visible for a range of networks, from having a sensitivity of 1 to the point where 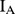 is maximized. In this region, the two quantities are weakly correlated and attractors can be almost arbitrarily robust to perturbations without restricting the propagation of information in the network.

## Introduction

Biological regulatory networks, such as gene regulatory networks (GRN), must balance the need to respond appropriately to incoming stimuli with the need to maintain robust behavior. Namely, the network must be able to distinguish and respond appropriately to various environmental and internal signals. At the same time, it cannot be so responsive that the input signals completely alter the behavior of the network. Several studies, using Random Boolean Networks (RBNs) as models of GRNs [1], [2], have investigated what topologies and logic transfer functions allow these networks to exhibit such properties [3]–[9].

Boolean networks (BN) have long been used to study global dynamic properties of biological networks, particularly as models of GRNs [2]. When used in this context, each gene (also called node) is represented as a Boolean variable. There is a clock that determines when the state of the nodes is updated. In synchronous networks, all nodes are synchronously updated at every time step, according to a Boolean function and the current state of its input nodes. Also, since BNs are deterministic and their state space is finite, it follows that a state must eventually repeat, after which the network will repeat a fixed sequence of states *ad infinitum*. These state cycles are the ‘attractors’ of the BN [2]. In the context of GRNs, it has been hypothesized that the attractors correspond to the different cell types that the network can express [2].

BNs exhibit a rich range of dynamics. In particular, groups of randomly generated of BNs (so-called “ensembles”) fall into two broad dynamical regimes: order and chaos [2]. The dynamic regime of the network can be determined from its response to perturbations, i.e., a deviation from the behavior prescribed by the topology and logic of the network. In general, ordered RBNs are very robust to perturbations – they behave similarly to the unperturbed network – however they lack the ability to respond differently to different perturbations [2], [10]. BNs in the chaotic regime tend to magnify small perturbations until the entire behavior of the network changes. Due to that, their responses to very similar perturbations differ widely. BNs operating in the threshold between order and chaos, called ‘critical’, exhibit a mix of both properties. They are robust to many perturbations, but not to a subset of the possible perturbations. Due to this ability to respond to specific perturbations, it has been proposed that biological networks operate in this regime [2]. Studies of the logic, topology, and response to perturbations of the yeast GRN have supported this hypothesis [4], [11], [12].

The most common method to measure the response to perturbations of a BN is to assess the number of nodes which are in a different state, one time step after a single-node perturbation, which is analogous to the Lyapunov exponent from continuous-time systems theory [10]. In BNs, this can be calculated analytically by the mean sensitivity of the Boolean functions to their inputs [5]. Networks with sensitivity less than 1 are said to be ordered since a perturbation of size 1 will shrink, on average, towards 0, while networks with sensitivity greater than 1 are said to be chaotic as the same initial perturbation will grow in size with each time-step. These assessments of robustness studied the response of RBNs to perturbations, starting from random states.

Here, we aim to study what properties of the GRN confer phenotypic robustness to the various cell types following differentiation, using RBNs as models of GRNs. Thus, we focus on the response to perturbations when the network is on an attractor since attractors correspond to the possible long-term behaviors of the network. However, to survive, cells need abilities other than phenotypic stability. One such property is the ability to propagate information between the genes of the network. E.g, signals both generated in the cell as well as arriving from the environment need to be processed so that a proper response can be generated.

One study has examined information propagation as a function of the BN’s dynamical regime, as quantified by the average mutual information (MI) between all pairs of nodes [3]. It was found that in the limit of infinite-sized networks, this quantity maximizes in the critical regime. In finite-sized networks, the maximization occurs slightly in the chaotic regime. Information propagation in RBNs has been also assessed by other methods, which also found a maximization at the critical regime [4], [6], [7], [9], [13]. A subsequent study has examined the distributions and properties of the MI between nodes while the network is on an attractor [14]. The distributions of MI within attractors (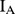) differ widely between networks in different dynamical regimes. The dynamic regime of the network, aside from affecting the 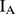, is also expected to affect the robustness of the attractors to perturbations, since this regimes affects for example, the size of the basins of attraction of the attractors [7], [15].

In a biological setting, we expect that both the ability of the network to process information and its robustness, i.e., its ability to remain in a confined region of the state space (even in the presence of noise [16]), are subject to selection. Selection ought to act on these two abilities at the level of each attractor that the network goes to, i.e., at the level of each cell type. It is unknown if these selective pressures are related, e.g., whether one constrains the other.

Here, we investigate the relationship between the information propagating in an attractor and its robustness to perturbations, depending on the dynamic regime of the network. First, we present the methods, after which we study the mean behavior of these properties as a function of the dynamic regime, followed by the correlation between them. Afterwards, we investigate the causes for observed relationships in each regime. In the end, we present our conclusions.

## Materials and Methods

In a RBN of N nodes, each node’s state at time *t* is a Boolean variable. The node’s state at the next time moment, *t*+1, is given by a Boolean function of a set of other nodes in the network. By iteratively applying this update rule, the Boolean network traces a trajectory through the state space. RBNs can be constructed by sampling from an ensemble of networks. For our purposes, we consider RBNs with exactly KN connections, which have been sampled at random without replacement from among all N^2^ possible connections, as in the Random 2 algorithm in [17]. The Boolean update rules have been sampled such that for each combination of input states, the output has a probability *p* of being 1, independent of all other outputs. The mean sensitivity, *s*, of these RBNs to single-node perturbations (the expected number of nodes that will change state due to changing the state of a single node) is 2K*p*(1−*p*) [5].

Attractor discovery was performed by initializing the network in a random state and running the network until a state repeated. For each network, 10^4^ random starting states were used, which is expected to be enough to find nearly all of the attractors of networks of our size (N = 50) [18]. Transient lengths were limited to 10^5^ states.

We then assess the robustness and 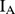
of the attractors. The robustness of an attractor, 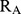, can be quantified by the fraction of the number of single-node perturbations from all states of the attractor after which the network returns back to the original attractor [19]. The size of a perturbation is the number of nodes whose state is perturbed at a moment in time. Here, we focus on single-node perturbations, that is, we assume that multiple simultaneous perturbations occur only on very long time scales, and can thus be ignored. Formally, 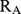, is defined as the probability that a trajectory starting from a state on an attractor with one node perturbed, will return to that attractor. This was calculated by perturbing each node in each state of an attractor, running the attractor search method described above starting with these states, and counting the fraction that returned to the original attractor.


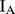
 is obtained from the average temporal mutual information between all pairs of nodes. The mutual information between two binary sequences of length *l* is defined based on their information entropy [20], which for a sequence *c* containing *p*_1_ *l* 1′s and *p*_0_ *l* 0′s is defined as:


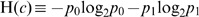
(1)

The entropy of the joint sequence between two sequences *c*_1_ and *c*_2_ with *p*_00_ *l* 0-0 pairs (with similar definitions for *p*_01_, *p*_10_ and *p*_11_) is defined as:


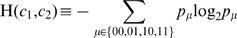
(2)

The mutual information between two binary sequences is then defined as:


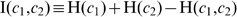
(3)

The mutual information between the timeseries of two nodes of a Boolean network, with the output timeseries shifted by one timestep (denoted by *c* ^1^), can be used as a measure of the amount of dynamical correlation between an input and an output. We use the average mutual information between all nodes as a measure of the average efficiency of information propagation in the network along that trajectory through state space [3]:


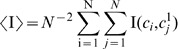
(4)

If a network runs long enough, the attractor it reaches will dominate the *p*’s used to calculate 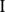. The average pairwise mutual information in the attractor (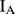) represents the average information propagation in one of the long-term behaviors of the network.

In strongly chaotic networks (*s*>2), the attractor discovery algorithm and robustness calculation become prohibitively expensive, since the amount of steps required to reach an attractor grows exponentially with sensitivity. For this reason, we opted to use networks with *p* = 0.75. These generally have smaller attractors and smaller transients than networks with the same sensitivity but with *p* closer to 0.5, while the Mutual Information is very similar to that of networks with the same sensitivity and *p* = 0.5 (Figure S1).

Finally, we study only networks with a mean sensitivity of 0.5 or greater, since below this value, instead of a network, there are only small, disconnected clusters of nodes.

## Results

**Figure 1:**
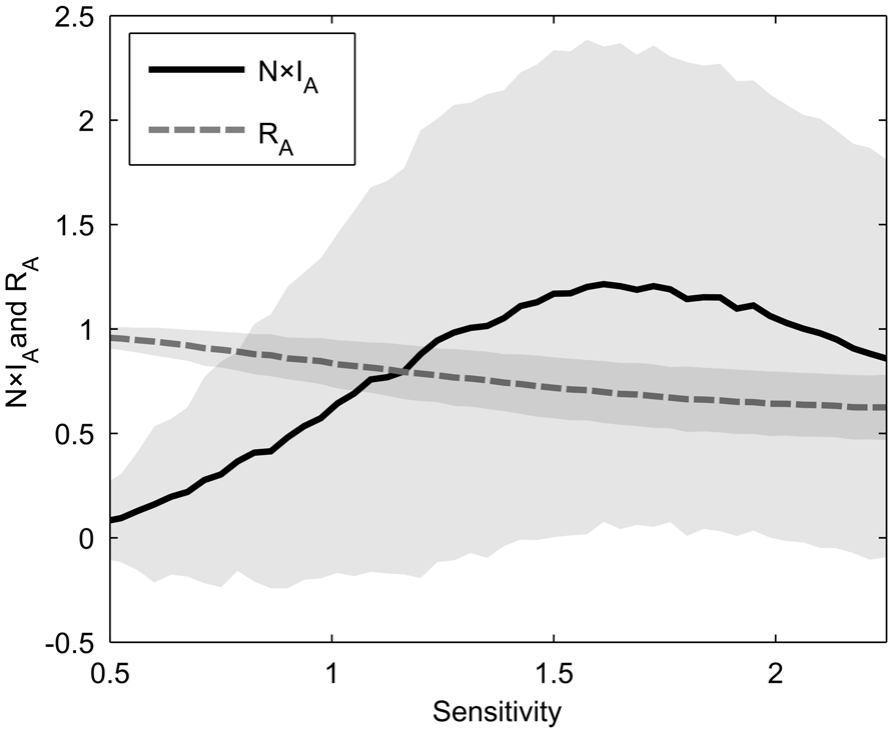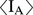 and 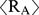 as a function of sensitivity. N = 50, *p* = 0.75, 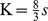, 10^4^ attractors were found per condition from at least 2000 networks generated randomly as described in Materials and Methods. Gray areas denote one standard deviation from the mean.

We first study how 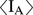 and 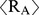 change with the sensitivity of network ensembles. For this, we simulated networks with 50 nodes, *p* = 0.75, with the sensitivity being adjusted by varying K. We searched for the attractors starting from 10^4^ random initial states, as described in Methods. Results are shown in Fig. 1. In agreement with previous results [14], 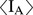 maximizes slightly in the chaotic regime, at sensitivity 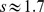, due to finite size effects. We note that the distributions of 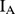 are heavy tailed, which is visible in the large standard deviation of the distribution (shown in grey), resulting in the relatively large sampling error considering the number of samples obtained (10^4^). Changes in the shape of these distributions with sensitivity are discussed in [14]. Meanwhile, 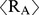 decreases monotonically with *s*.

To investigate the relationship between the information propagation within an attractor and its robustness, we calculated the correlation between the 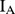 and 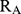 from 10^4^ attractors of networks with varying sensitivity. We opted to use Kendall’s τ rank correlation metric [21] rather than linear correlation since the relationship between 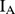 and 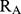 is not linear (Figure S2). Kendall’s τ calculates the fraction of pairs of (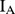,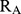) tuples which are consistent [21], i.e. increasing/decreasing 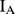 leads to increasing/decreasing 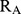, respectively.

**Figure 2:**
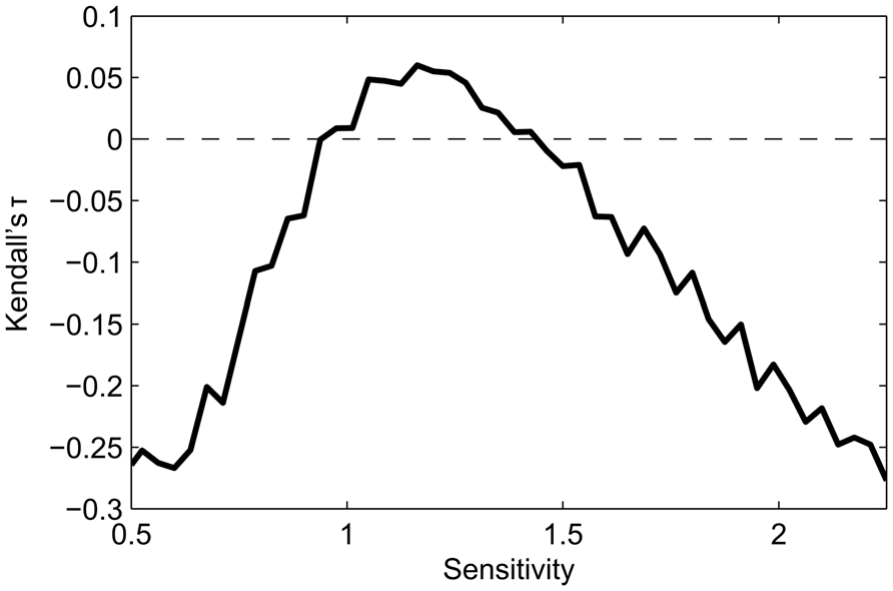
Kendall’s τ rank correlation between 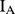 and 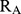. N = 50, *p* = 0.75, 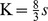, 10^4^ attractors were found per condition from at least 2000 networks generated randomly as described in Materials and Methods. For 10^4^ samples, |τ| values greater than 0.017 are considered significant (p-value <0.01).

In ordered and chaotic networks, 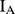 and 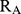 are found to be anti-correlated (Fig. 2), indicating that attractors that propagate information more efficiently (i.e. that make more pairs of nodes to be temporally correlated or make the fraction of correlated pairs to be more strongly correlated) are generally more susceptible to perturbations. This is in line with the intuition that an attractor capable of propagating information is equally capable to propagate a perturbation. Interestingly, there is a window of weak positive correlation starting from *s* = 1 slightly into the chaotic regime, returning to anti-correlation near the point where 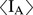 is maximized. We next focus on explaining the observed anti-correlation in the chaotic and ordered regimes.

In deeply chaotic networks (random maps), the notion of distance between states is meaningless. That is, two states that differ in only one node’s state are not necessarily “close” in the state space. Consequently, the chance that the network will return to a particular attractor after one node is perturbed while in that attractor is the same as the chance that any randomly chosen state leads to that attractor. This is, in turn, directly proportional to the size of the set of states from which the network will settle into that attractor (also known as the basin of attraction). Due to this, 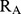 is correlated with basin size.

Further, the size of the basin of attraction is directly related to the length of the attractor, since each state of the attractor is expected to ‘drain’ a similar amount of basin states. This linear relationship was shown in [15]. The attractor length is anti-correlated with 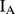 since, in this regime, 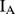 is spurious and thus is lower in longer sequences than shorter ones [22]. In summary, in the chaotic regime, 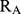 is correlated with basin size, which is correlated with attractor length, which is anti-correlated with 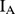, causing 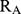 to be anti-correlated with 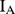.

In the ordered regime, there are two differences to the above reasoning. First, the basin size is anti-correlated with attractor length [15]. Second, point attractors (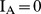) become prevalent. Attractors with multiple states generally have positive, non-spurious 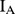 and, in this regime, attractor length and 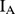 are positively correlated. As a result, in the ordered regime, 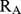 and 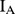
are negatively correlated.

**Figure 3:**
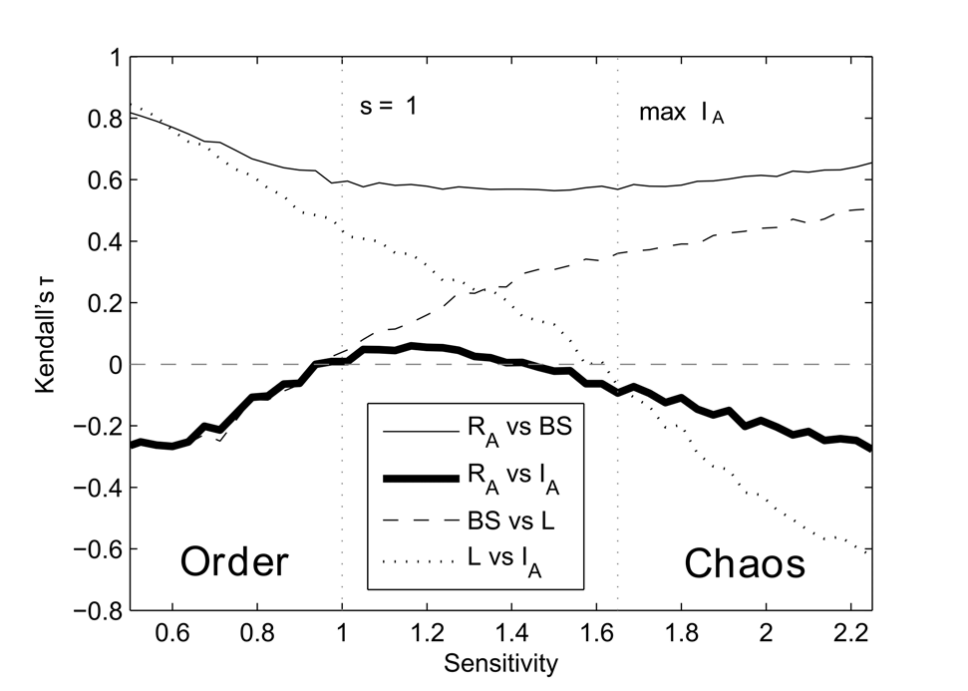
Kendall’s τ rank correlation between 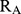, the size of the basin of attraction (BS), the length of the attractor (L) and 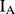. N = 50, *p* = 0.75, 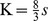, 10^4^ attractors were found per condition from at least 2000 networks generated randomly as described in Materials and Methods. For 10^4^ samples, |τ| values greater than 0.017 are considered significant (p-value <0.01).

Two of the above relationships change sign from the ordered to the chaotic regime. For each of these relationships, there will necessarily be a point of no-correlation. To test this, we plot the pairwise relationships between 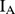, 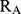, and the properties above, specifically basin size (BS) and attractor length (L) (Figure 3). The correlation between basin size and attractor length is zero at *s* = 1, as previously reported in [15]. The correlation between attractor length and 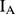 is zero almost exactly at the point where mean 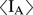 maximizes due to finite size effects [3]. These points correspond with the points of no correlation between 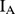 and 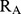.

The region of weak, positive correlation between 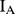
and 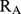 is also predictable from the chain of correlations described above, as all of these are positive in this region. Relevantly, the fact that 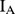 and 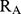 are *weakly* correlated implies that, for critical networks, attractors can have a high value of 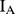 with no strong restrictions on the value of 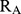. Outside this region, it is unlikely, e.g., to find an attractor with both high 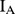 and high 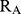.

Since the sensitivity value for which 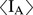 maximizes depends on the size of the network [3], we expect the size of the window of weak positive correlation to shrink in larger networks and grow in smaller networks. To test this, we recreated Figure 3 with N = 25 and found that this prediction holds (Figure S3).

### Conclusions

We tested for a relationship between the robustness to perturbations of an attractor in a RBN and the amount of information propagated within the network when in that attractor. By demonstrating that a chain of correlations exist between certain properties of the attractors, we found that in the ordered and chaotic regimes, these quantities are anti-correlated. That is, there is a trade-off between robustness and information propagation. At the boundary between order and chaos (*s* = 1), robustness is not correlated with information propagation. Further, for finite size networks, there is a small parameter range, starting at *s* = 1 and extending into chaos, where robustness is weakly, positively correlated with information propagation. This window extends to the point of maximal information propagation, and its size decreases with network size.

## Discussion

From a single totipotent cell, via the processes of cell division and differentiation, an organism is formed that consists of multiple, widely distinct cell types. Since gene expression and most other molecular mechanisms underlying this process are stochastic [23], there is a need for multiple mechanisms, such as chromatin remodeling, which constrain the kinetics of individual genes of fully differentiated cells, since the patterns of expression levels of the genes of these cells is clearly within very constrained regions of the state space [24], [25].

One key property that constrains gene’s expression levels is the existence of multiple gene-gene interactions that form a regulatory network. This network of interactions constrains their activities via inhibitory or excitatory relationships. It is likely that throughout the evolutionary process, these networks evolved towards, to some extent, make differentiated cell types as phenotypically robust as possible. Malfunctioning of this stability may be one of the underlying causes of diseases such as cancer [26].

Aside from being responsible for the stability of differentiated cells, this network of interactions between genes is also responsible for the propagation of information between genes. This flow of information is essential for survival. For example, some genes express proteins that are responsible for the detection of specific external signals, and following this detection, activate other genes which will express proteins that will be responsible for a proper response of the cell.

Provided that cellular fitness depends on such coordination of genes’ activities, maximization of this coordinated flow of information is likely to be selectively advantageous. To the extent that RBN models capture the essential features of the organization of genetic networks, we found that critical networks are naturally favored as they allow maximizing this flow of information, without compromising robustness of its attractors to perturbations.

As a final remark, it needs to be stated that real genetic networks are not random, as they were shaped by selection. Also, it is possible that there are networks with a specific topology and logic of the transfer functions such that, while classified as ordered or chaotic, would also exhibit high capacity for information transfer between the genes and, simultaneously, strong robustness to perturbations. What our results indicate is that networks with these properties are far rarer in these regimes than in the critical regime. Given this, and since mutations are, seemingly, random, it is thus more likely that the evolutionary process has reached these favorable features by selecting for networks in, or near, the critical regime.

## Supporting Information

Figure S1Mean N 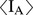 as a function of sensitivity for various network sizes (N). *p* = 0.75, 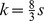, 10^4^ networks were generated for each condition and one attractor was sampled.(TIF)

Figure S2Joint distribution of 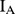 and 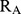 from attractors sampled from 5000 networks *p* = 0.75, k = 4 (*s* = 1.5).(TIF)

Figure S3Kendall’s τ rank correlation between 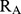, the size of the attractor’s basin of attraction (BS), the length of the attractor (L) and 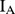. N = 25, p = 0.75, 10^4^ attractors were found per condition from at least 2000 networks.(TIF)
