# Supplementary figures and images for: Robustness and Information Propagation in Attractors of Random Boolean Networks
Source: PLoS One. 2012 Jul 30;7(7):e42018. doi: 10.1371/journal.pone.0042018 (PMC3408442; doi:10.1371/journal.pone.0042018)

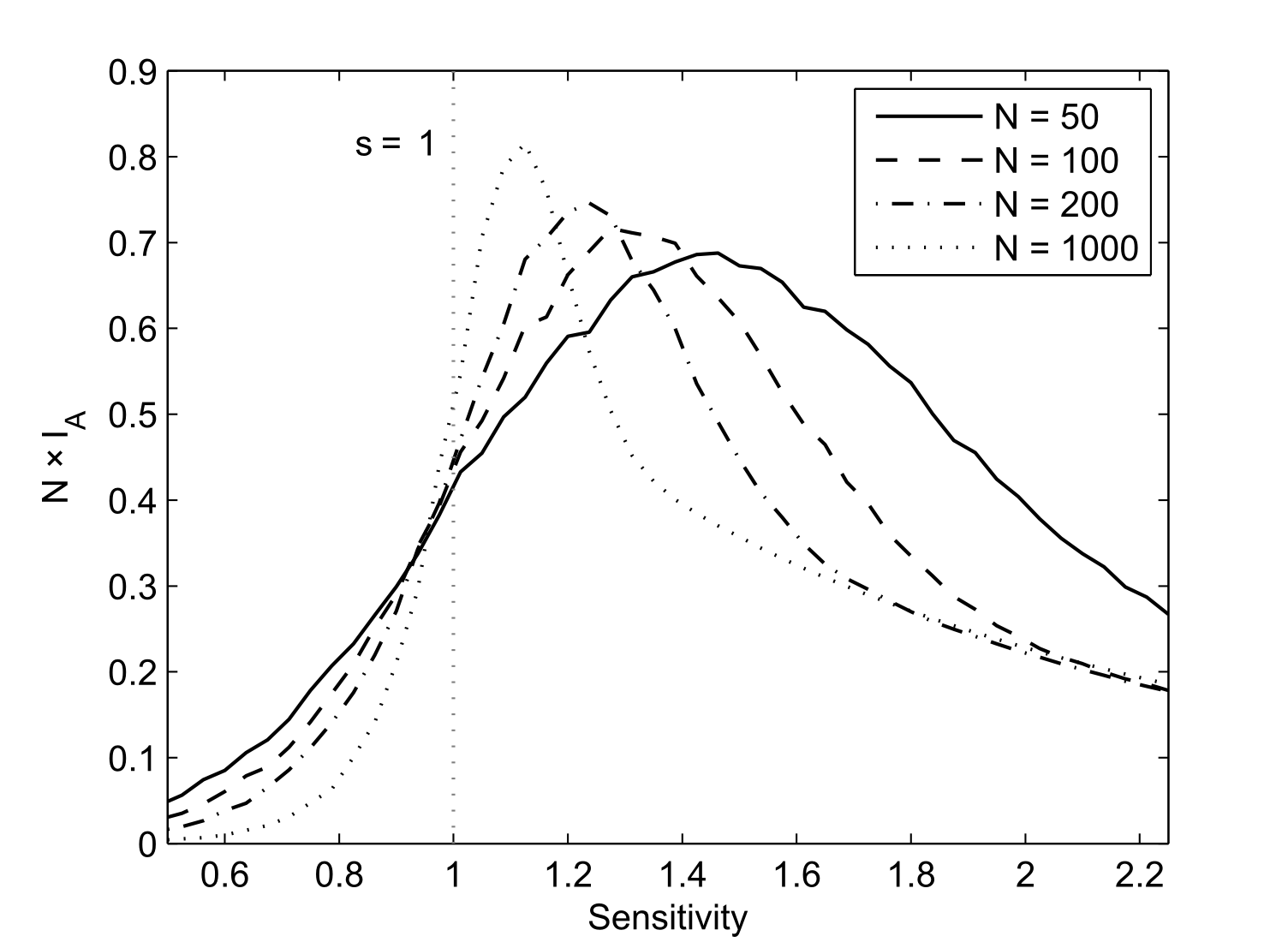

Supplement: Figure S1 — Mean N as a function of sensitivity for various network sizes (N). p = 0.75, , 104 networks were generated for each condition and one attractor was sampled. (TIF) [file pone.0042018.s001.tif]

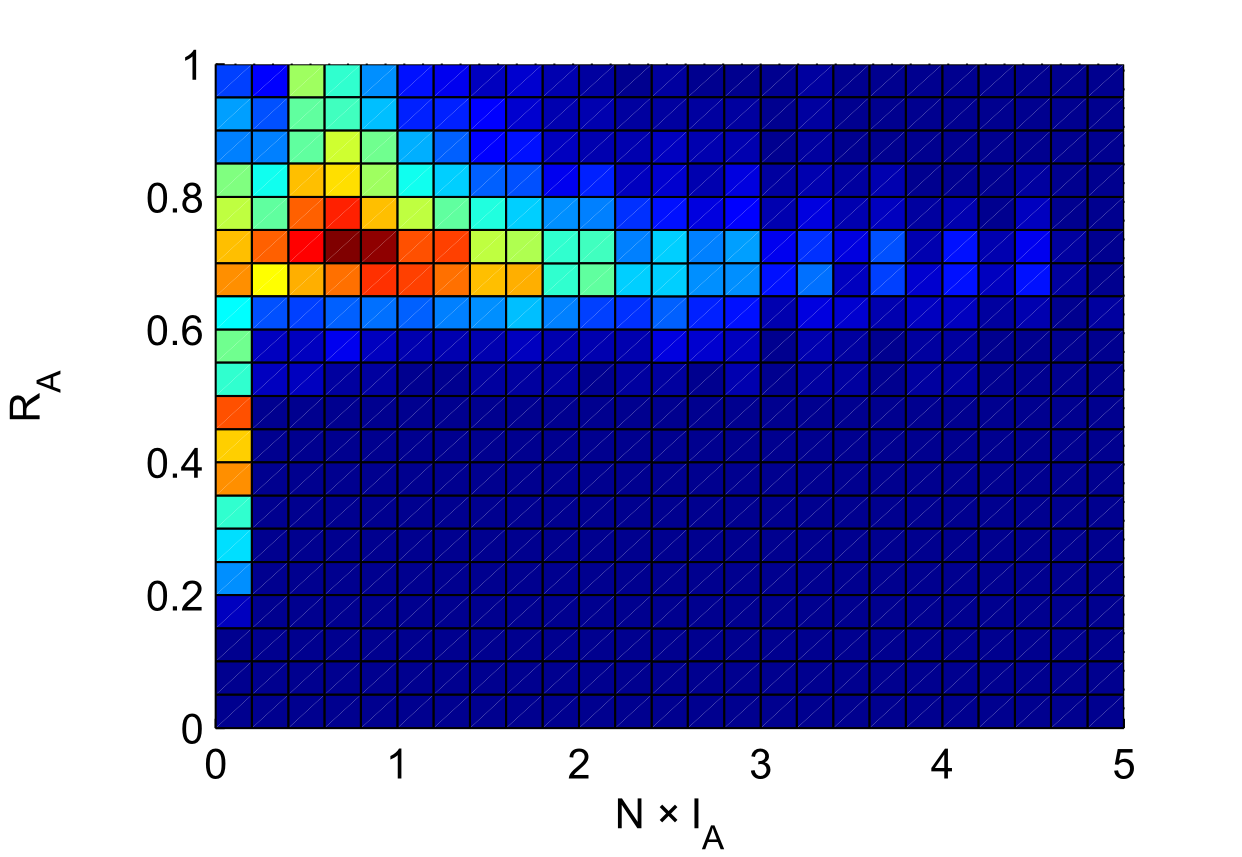

Supplement: Figure S2 — Joint distribution of and from attractors sampled from 5000 networks p = 0.75, k = 4 (s = 1.5). (TIF) [file pone.0042018.s002.tif]

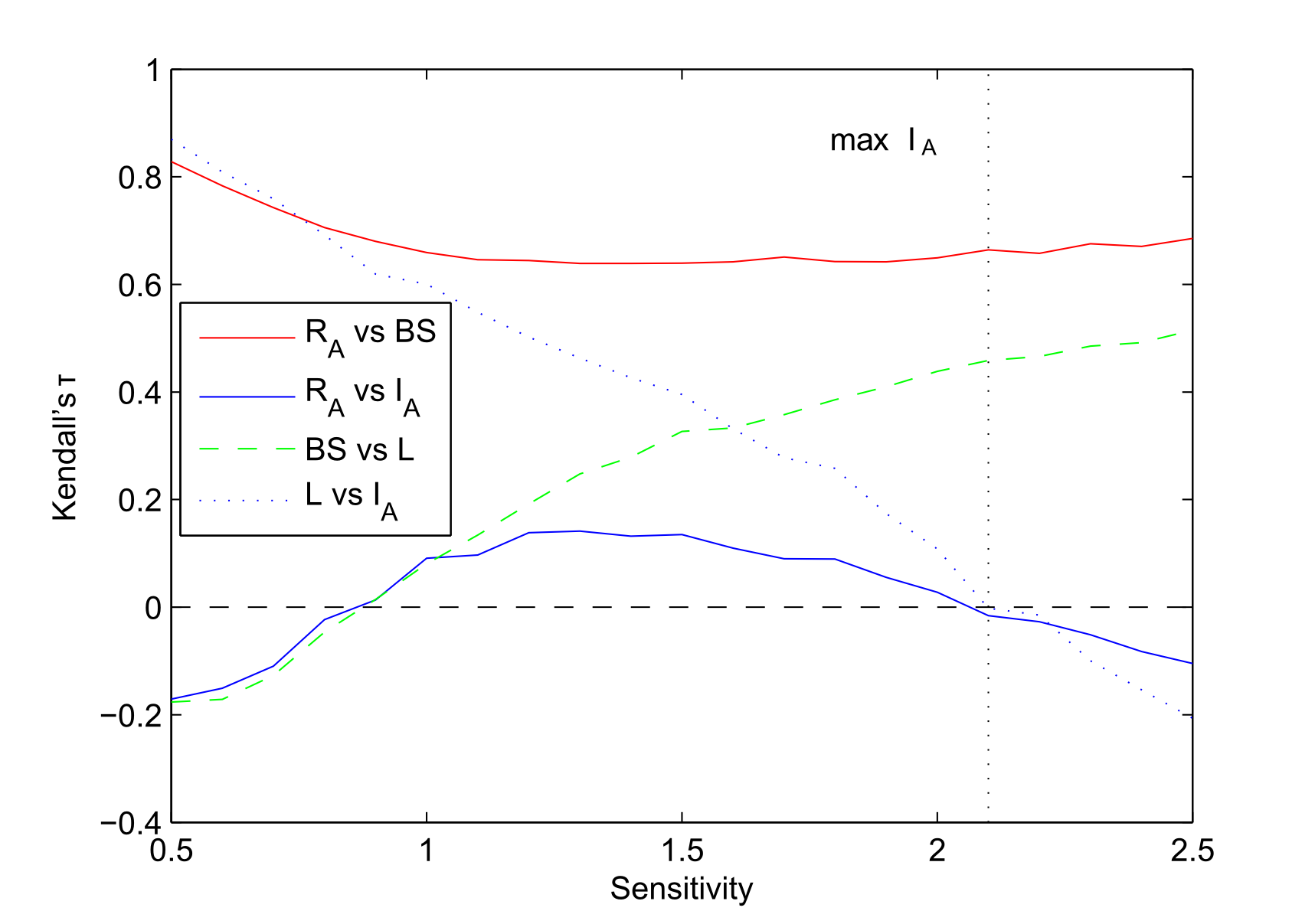

Supplement: Figure S3 — Kendall’s τ rank correlation between , the size of the attractor’s basin of attraction (BS), the length of the attractor (L) and . N = 25, p = 0.75, 104 attractors were found per condition from at least 2000 networks. (TIF) [file pone.0042018.s003.tif]
